# Conservative versus surgical management of pediatric bipartite patella with patellar dislocation: a single-center retrospective analysis

**DOI:** 10.3389/fsurg.2026.1738416

**Published:** 2026-01-29

**Authors:** Xipeng Wang, Kun Nie, Jiangtao Liu

**Affiliations:** Department of Orthopedic Surgery, the Central Hospital of Wuhan, Tongji Medical College, Huazhong University of Science and Technology, Wuhan, China

**Keywords:** bipartite patella, lateral retinacular release, MPFL reconstruction, patellar dislocation, pediatric orthopedics

## Abstract

**Objective:**

To evaluate clinical features and compare outcomes of conservative vs. surgical management in pediatric patients with congenital bipartite patella (BP) and patellar dislocation.

**Methods:**

A retrospective study included 42 children (≤16 years) with BP and patellar dislocation treated between January 2019 and January 2024. Patients received either conservative treatment (*n* = 18; reduction, immobilization, and rehabilitation) or surgical treatment [*n* = 24; lateral retinacular release, medial patellofemoral ligament (MPFL) reconstruction, ± internal fixation of accessory fragments]. Functional outcomes were assessed using Lysholm, Kujala, and visual analog scale (VAS) scores preoperatively and at final follow-up. Radiological parameters included patellar congruence angle, tibial tubercle–trochlear groove distance, and accessory fragment healing. Complications and recurrence rates were recorded.

**Results:**

Baseline characteristics were comparable, except for a higher proportion of having suffering recurrent dislocations in the surgical group (*P* = 0.001). Both groups improved significantly in functional scores (*P* < 0.05), but the surgical group demonstrated superior outcomes (Lysholm: 90.5 ± 4.8 vs. 76.3 ± 8.1; Kujala: 92.1 ± 3.9 vs. 78.9 ± 7.5; VAS: 1.2 ± 0.9 vs. 3.1 ± 1.4; *P* < 0.001). Surgical patients achieved normalization of patellofemoral alignment, and all eight patients undergoing internal fixation achieved solid bony union. Recurrence occurred in 27.8% of conservatively treated patients but in none of the surgical group. No major complications were reported.

**Conclusion:**

In pediatric BP with patellar dislocation, surgical management combining MPFL reconstruction and lateral retinacular release, with selective internal fixation, provides superior functional recovery, pain relief, and lower recurrence compared to conservative therapy, suggesting that surgical management may provide superior outcomes in selected pediatric patients.

## 1 Background

Patellofemoral joint instability is one of the common knee disorders in children and adolescents, with diverse etiological factors including bony structural abnormalities and soft tissue imbalances. Congenital bipartite patella (BP) is a rare patellar developmental variant, first systematically described by Saupe in 1921. It is characterized by two or more ossification centers that fail to fully fuse. Based on the location of accessory bone fragments, Saupe classified it into three types: Type I, Type II, and Type III (1). In most cases, BP is asymptomatic and is often incidentally discovered during knee x-ray examinations for other reasons. However, the clinical management becomes particularly complex when BP dislocation coexists. Traumatic mechanisms or chronic instability of patella dislocation may induce micromotion or separation at the fibrocartilaginous junction of the BP, leading to symptoms such as pain and locking, a condition known as “symptomatic BP” (2, 3). In pediatric patients, where bones are still developing and growth plates remain active, treatment strategies require extreme cautions. These must address the underlying patellar instability while addressing symptomatic accessory bone fragments, all while avoiding damage to the growth plate.

Although substantial literature exists regarding either isolated BP or isolated patellar dislocation, systematic investigations that integrate these two conditions as a single clinical entity remain scarce, particularly within pediatric populations. The optimal management approach, whether conservative treatment or surgical intervention, continues to be a matter of debate. Among surgical strategies, no consensus has been established regarding the necessity of excising the accessory fragment with internal fixation vs. performing concomitant patellofemoral stabilization procedures, such as medial patellofemoral ligament (MPFL) reconstruction (4). To address this knowledge gap, we conducted a retrospective analysis of 42 pediatric patients with congenital BP accompanied by patellar dislocation treated at our institution. This study aimed to delineate the clinical characteristics and compare therapeutic outcomes, thereby providing evidence-based recommendations and systematic guidance for clinical decision-making. Therefore, systematic investigation of pediatric BP with patellar dislocation is warranted to guide optimal management.

## 2 Methods

### 2.1 Clinical data

#### 2.1.1 Subjects

A retrospective study was conducted to consecutively enroll pediatric patients diagnosed with congenital BP accompanied by patellar dislocation who were treated in the Department of Orthopedics at our hospital between January 2019 and January 2024. The study protocol was reviewed and approved by the Institutional Ethics Committee of our hospital.

#### 2.1.2 Inclusion criteria

(1) Age ≤16 years; (2) Diagnosis of congenital BP (Saupe Type II or III) confirmed by x-ray, CT, or MRI with acute or recurrent patella dislocation; (3) Availability of complete clinical data, including detailed medical history, physical examination findings, and imaging studies; (4) Minimum follow-up duration of 18 months.

#### 2.1.3 Exclusion criteria

(1) Acute traumatic patellar fracture; (2) Patellar dislocation secondary to neuromuscular disorders or syndromic conditions; (3) Concomitant major ligamentous injuries of the knee joint (e.g., anterior or posterior cruciate ligament injuries); (4) Loss to follow-up or incomplete clinical data.

### 2.2 Treatment methods

#### 2.2.1 Grouping

Patients were grouped according to the initial treatment strategy rather than the type of dislocation. Based on the patient's clinical presentation, family preference, and comprehensive evaluation by the attending physicians, 42 children were categorized into two groups: (1) Conservative Treatment Group (*n* = 18): This group primarily included children with acute primary dislocation who achieved successful patellar reduction, exhibited minimal tenderness over the accessory fragment, and demonstrated no significant fragment separation on imaging (gap <2 mm) and those whose parent(s) decided to be conservatively treated; (2) Surgical treatment group (*n* = 24): This group mainly consisted of children with recurrent dislocation, failure of conservative management (persistent pain or instability), or radiographic evidence of significant separation of the accessory bone fragment (≥2 mm) accompanied by persistent local tenderness.

#### 2.2.2 Conservative treatment plan

(1) Acute phase management: For patients with acute dislocation, immediate manual reduction was performed, followed by immobilization of the knee in full extension using a knee brace or long-leg plaster splint for 4–6 weeks. (2) Rehabilitation phase: After completing the immobilization period, a structured rehabilitation program was initiated. The regimen emphasized quadriceps strengthening—particularly targeting the vastus medialis oblique (VMO)—along with knee joint range-of-motion exercises and proprioceptive training. Patients and their guardians were instructed to avoid strenuous activities and movements that could predispose to recurrent dislocation.

#### 2.2.3 Surgical treatment

**Figure 1:**
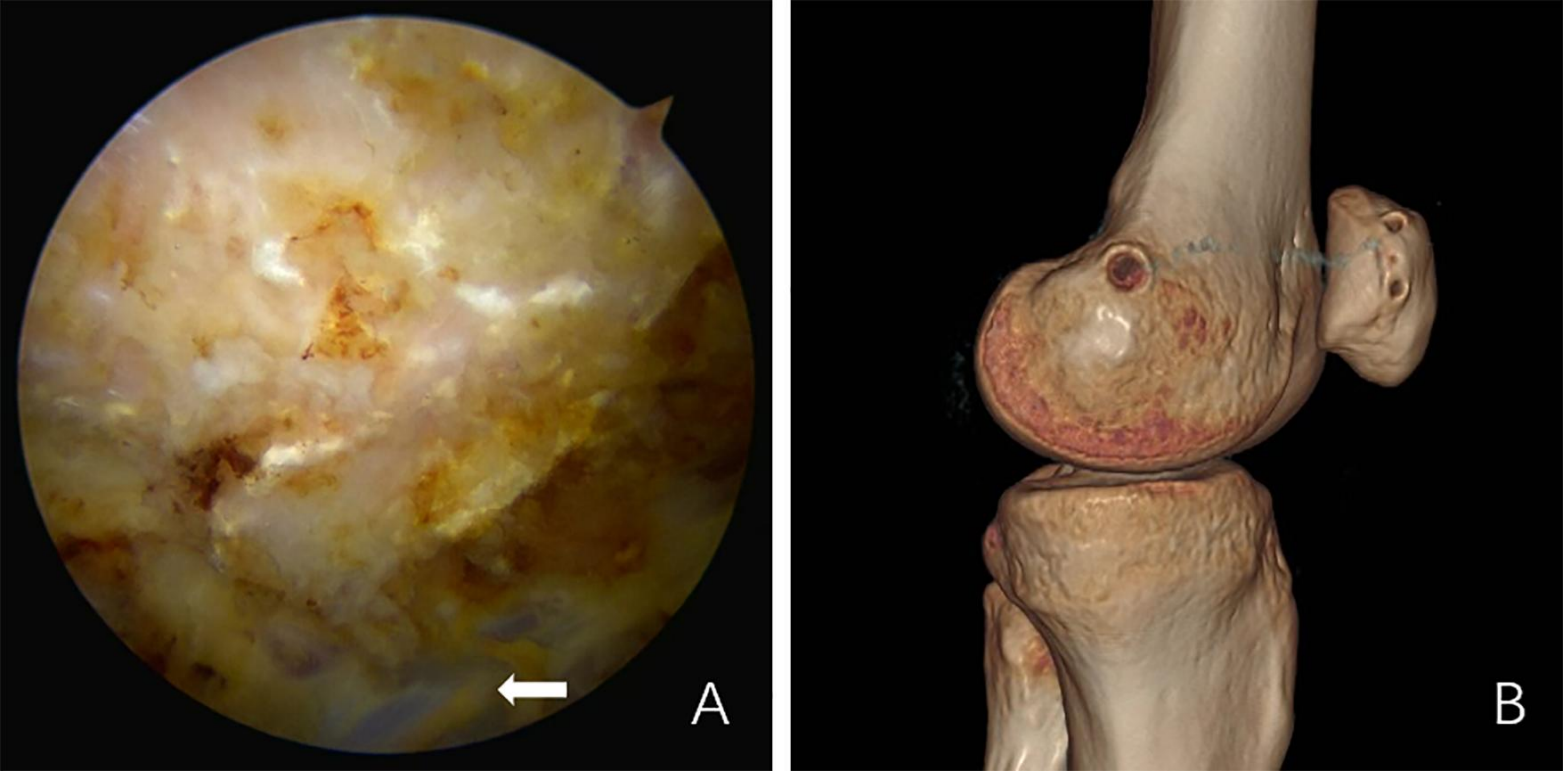
MPFL reconstruction. **(A)** Intraoperative arthroscopic anchor fixation of the semitendinosus tendon (arrow). **(B)** Postoperative CT 3D reconstruction shows the position of the anchors in the medial groove of the patella and the medial femoral condyle.

(1) All surgical procedures were performed under general anesthesia by the same team of senior orthopedic surgeons, with the use of a pneumatic tourniquet to ensure hemostasis. (2) Surgical approach: An anteromedial knee incision was primarily used; in some cases, it was combined with a small auxiliary lateral incision for improved exposure. (3) Key steps of the operation: (a) Arthroscopic examination: Diagnostic arthroscopy was first conducted to assess intra-articular structures, including the articular cartilage, meniscus, and patellar tracking. (b) Collateral band release: A complete release of the lateral retinaculum was performed in all patients to alleviate excessive lateral tension on the patella and facilitate proper realignment. (c) Medial patellofemoral ligament (MPFL) reconstruction: The patient's autologous semitendinosus tendon was harvested and prepared using a double-strand technique. The superior aspect of the patellar accessory bone fragment was resected with an osteotome to expose the medial border at the midpoint and upper-third junction of the main patellar fragment, serving as the attachment site for the reconstructed MPFL. The graft was then fixed anatomically, and arthroscopic confirmation was performed to ensure proper patellar alignment, absence of tilt, and appropriate graft tension (Figure 1). (d) Internal fixation of accessory bone fragments: For smaller accessory fragments, particularly those affecting MPFL reconstruction, excision was performed. For larger fragments, however, contouring followed by screw compression fixation was performed. (4) Postoperative management: Postoperatively, all patients were fitted with an adjustable knee brace. During the early rehabilitation phase (0–2 weeks), passive range-of-motion exercises were initiated, followed by gradual progression to active mobilization between 2 and 6 weeks. Progressive weight-bearing and muscle strengthening commenced after 6 weeks, with gradual return to sports activities within 3–6 months depending on individual recovery progress.

#### 2.2.4 Criteria

(1) Functional score: All children completed the following scores before treatment and at the last follow-up: (a) Lysholm knee joint score: to evaluate the daily function of the knee joint (total score of 100 points, excellent ≥95, good 84–94, medium 65–83, poor <65). (b) Kujala patellofemoral joint score: a specific assessment of patellofemoral joint function (out of 100 points, the higher the score, the better the function). (c) Visual analog score (VAS): Assess the degree of pain at rest and exercise (0 for no pain, 10 for severe pain). (2) Radiological evaluation: preoperative and final follow-up imaging assessments included standard anteroposterior and axial (Merchant) radiographs, as well as CT and MRI scans. The following parameters were measured and analyzed: (a) Patellar congruence angle (CA): Normal range: 6° ± 11°, with angles exceeding 16° considered abnormal. (b) Femoral sulcus angle (SA): Normal value is 138 ± 6, >150 indicates dysplasia of the trochlea. (c) Tibial tubercle–trochlear groove (TT–TG) distance: measured on CT, >15–20 mm is abnormal. (d) Accessory bone status: Evaluated based on the separation between the accessory and main patellar fragments, presence of sclerotic margins, and evidence of fragment healing.

#### 2.2.5 Complications and recurrence rate

All perioperative and postoperative complications were systematically recorded for both groups throughout the treatment and follow-up periods. Documented complications included surgical site infection, neurovascular injury, internal fixation failure, and recurrent patellar instability (redislocation or subluxation). The recurrence rate of patellar dislocation was calculated and compared between the two groups.

### 2.3 Statistical analysis

All statistical analyses were performed using SPSS version 26.0 (IBM Corp., Armonk, NY, USA). Quantitative variables were expressed as mean ± standard deviation (mean ± SD). Intra-group comparisons were analyzed using paired *t*-tests, while inter-group comparisons were performed using independent-samples *t*-tests. Categorical variables were presented as frequencies and percentages, and inter-group differences were evaluated using the chi-square (χ^2^) test or Fisher's exact test where appropriate. A *P*-value <0.05 was considered statistically significant.

## 3 Results

### 3.1 Baseline characteristics

A total of 42 pediatric patients with congenital BP and patellar dislocation were included in the study, comprising 25 males and 17 females, with ages ranging from 8 to 16 years (mean ± SD: 11.2 ± 1.8 years). The affected side involved the left knee in 23 cases and the right knee in 19 cases. According to the Saupe classification, 9 patients were classified as Type II and 33 as Type III. All patients had a documented history of patellar dislocation, including 20 cases of acute primary dislocation and 22 cases of recurrent dislocation.

A comparative analysis of baseline data between the conservative and surgical treatment groups is presented in Table 1. No significant differences were observed in age, sex distribution, laterality, Saupe classification, or follow-up duration between groups (*P* > 0.05). However, a significantly higher proportion of recurrent dislocations was noted in the surgical treatment group compared with the conservative group (*P* = 0.001).

**Table 1:** Comparison of baseline data between groups.

Variable	Total (*n* = 42)	Conservative treatment group (*n* = 18)	Surgical treatment group (*n* = 24)	*t*/χ^2^-value	*P*-value
Age (years)	11.2 ± 1.8	10.8 ± 1.9	11.5 ± 1.7	1.302	0.201
Gender (Male/Female)	25/17	10/8	15/9	0.152	0.697
Laterality (Left/Right)	23/19	10/8	13/11	0.009	0.925
Saupe classification (II/III)	9/33	5/13	4/20	0.581	0.446
Type of dislocation (Primary/Recurrent)	20/22	14/4	6/18	10.286	0.001
Time from injury to visit (months)	5.6 ± 4.1	4.2 ± 3.0	6.6 ± 4.5	1.987	0.054
Follow-up time (months)	28.5 ± 6.2	27.8 ± 5.9	29.0 ± 6.5	0.642	0.525

### 3.2 Functional assessments

Preoperative evaluation showed no significant differences between the conservative and surgical treatment groups in Lysholm score, Kujala score, or VAS score (*P* > 0.05 for all), indicating comparable baseline knee function and pain. At the final follow-up, both groups demonstrated significant improvements in all functional scores compared to their respective preoperative values (*P* < 0.001).

**Figure 2:**
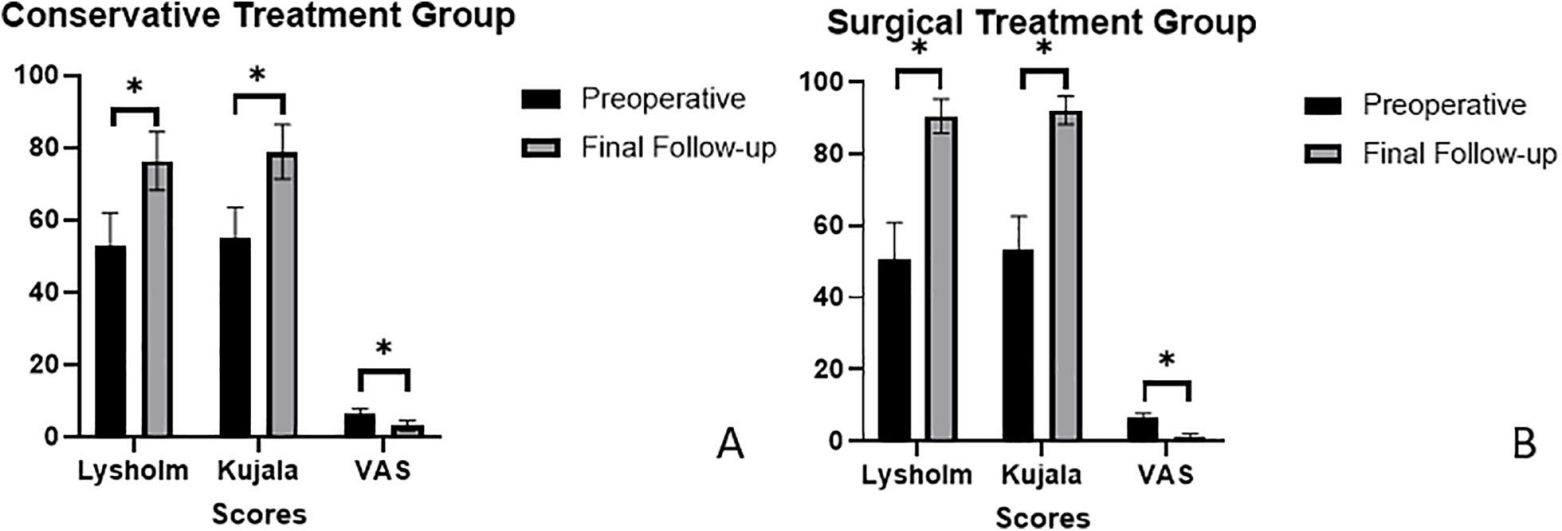
Bar charts comparing preoperative and final follow-up scores in conservative and surgical treatment groups. Both groups show improved Lysholm, Kujala, and VAS scores at follow-up, with significant improvements indicated by asterisks (p < 0.001). **(A)** Scores of conservative treatment group. **(B)** Scores of surgical treatment group.

At final follow-up, inter-group comparisons showed that the surgical treatment group had significantly higher Lysholm and Kujala scores than the conservative treatment group, while VAS scores were significantly lower (*P* < 0.001), indicating superior functional recovery and pain relief in the surgical cohort (Table 2 and Figure 2).

**Table 2:** Comparison of functional scores between groups before and after surgery.

Scores	Conservative treatment group (*n* = 18)		Surgical treatment group (*n* = 24)	
	Preoperative	Final Follow-up	Preoperative	Final follow-up
Lysholm score	52.6 ± 9.4	76.3 ± 8.1*	50.8 ± 10.1	90.5 ± 4.8*
Kujala score	54.8 ± 8.7	78.9 ± 7.5*	53.1 ± 9.5	92.1 ± 3.9*
VAS score	6.2 ± 1.5	3.1 ± 1.4*	6.5 ± 1.3	1.2 ± 0.9*

* *P* < 0.001.

### 3.3 Imaging comparison at final follow-up

#### 3.3.1 Patellar stability

At the final follow-up, all patients in the surgical treatment group demonstrated stable patellar tracking without any recurrence of dislocation or subluxation (recurrence rate: 0%). In contrast, 5 patients in the conservative treatment group (5/18, 27.8%) developed recurrent patellar instability, including three cases of subluxation and two cases of redislocation, all of which ultimately required surgical intervention.

#### 3.3.2 Patellofemoral alignment parameters

In the surgical treatment group, significant postoperative improvements were observed in the congruence angle (CA) and tibial tubercle–trochlear groove (TT–TG) distance. The mean CA decreased from 25.6° ± 8.1° preoperatively to −2.1° ± 5.3° at the final follow-up, and the TT–TG distance decreased from 18.5 ± 3.2 mm to 11.2 ± 2.1 mm (*P* < 0.01 for both). Although similar trends of improvement were noted in the conservative treatment group, their final values remained outside the normal physiological range, indicating suboptimal correction of patellofemoral malalignment.

#### 3.3.3 Accessory bone healing

Among the surgical cases, all 8 patients who underwent internal fixation achieved solid bony union, while the 16 patients who underwent accessory fragment resection exhibited satisfactory bone remodeling without local osteophyte formation. In contrast, 3 patients in the conservative treatment group showed persistent widening of the intercondylar gap accompanied by localized pain, suggesting incomplete bone healing or fibrous union.

#### 3.3.4 Representative imaging findings

**Figure 3:**
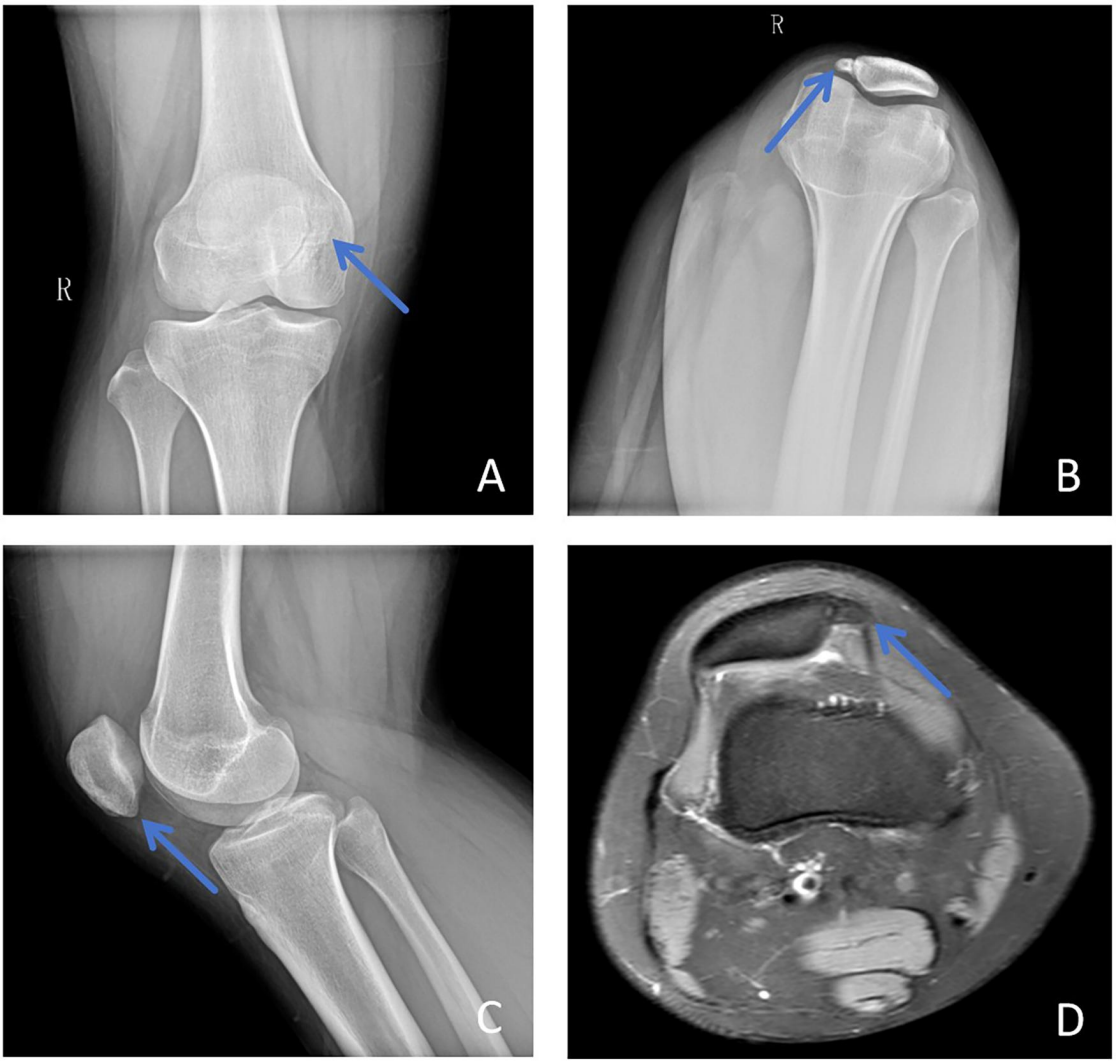
Preoperative imaging. **(A)** Anteroposterior view of the right patella. **(B)** Axial view of the right patella. **(C)** Lateral view of the right patella. **(D)** MRI of the right knee joint. On the anteroposterior and axial x-rays of the right patella **(A,B)**, the cortical bone appears continuous and smooth, with a nodular high-density lesion adjacent to the patella. The lesion exhibits well-defined margins, consistent with a diagnosis of BP. The left patella shows no abnormalities on anteroposterior or lateral views **(C)** MRI of the right knee **(D)** demonstrates a high-riding patella with slight lateral displacement, accompanied by a small nodular lesion on the medial aspect exhibiting low signal intensity on both T1- and T2-weighted images.

**Figure 4:**
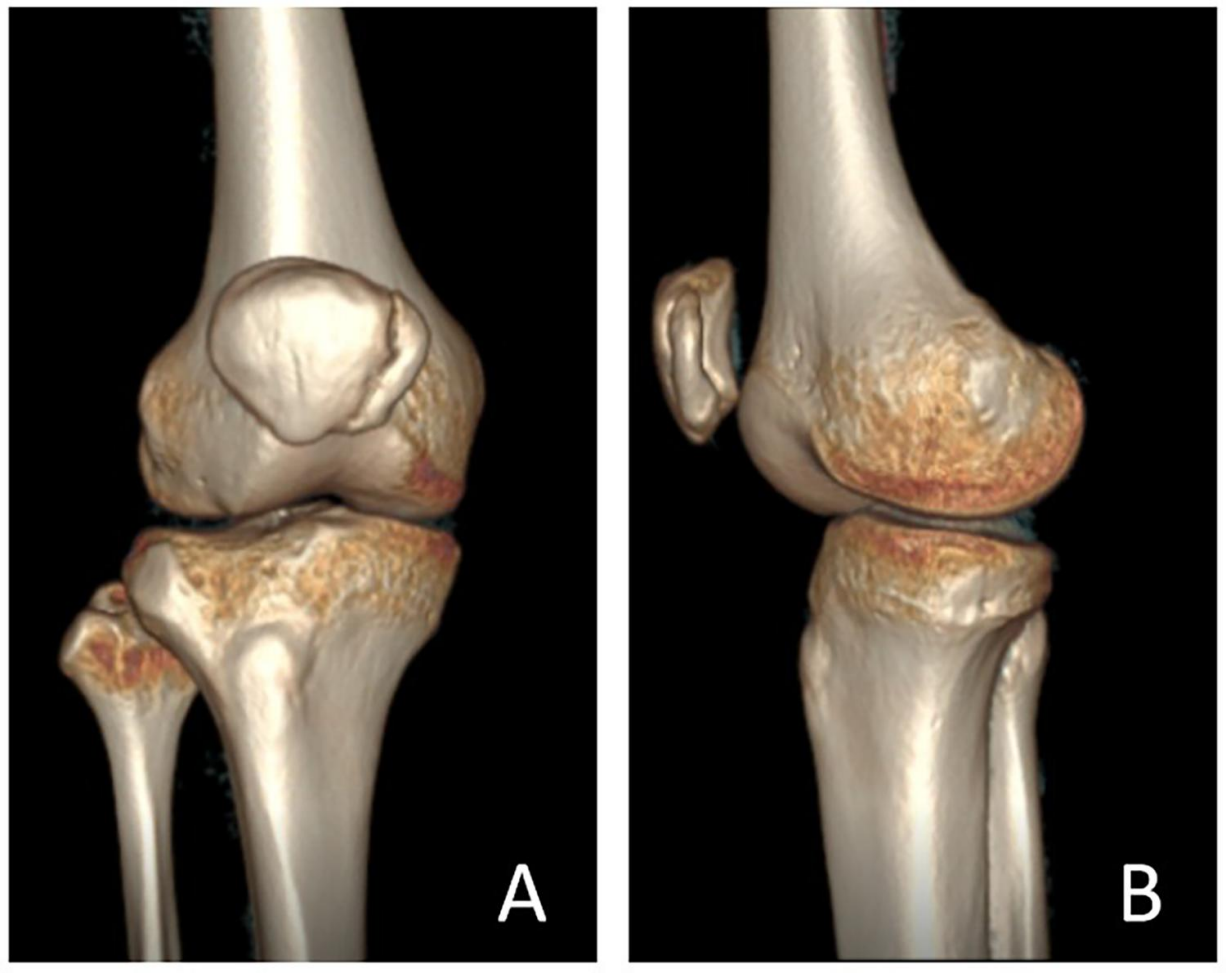
Preoperative right knee CT scan with 3D reconstruction. CT imaging and three-dimensional reconstruction of the right knee demonstrate lateral displacement of the right patella, with a bony high-density lesion located medially. No abnormal bony signal is observed in the proximal tibia, proximal fibula, or distal femur. These findings are consistent with BP of the right knee with patella alta. **(A)** Frontal view. **(B)** Lateral view.

**Figure 5:**
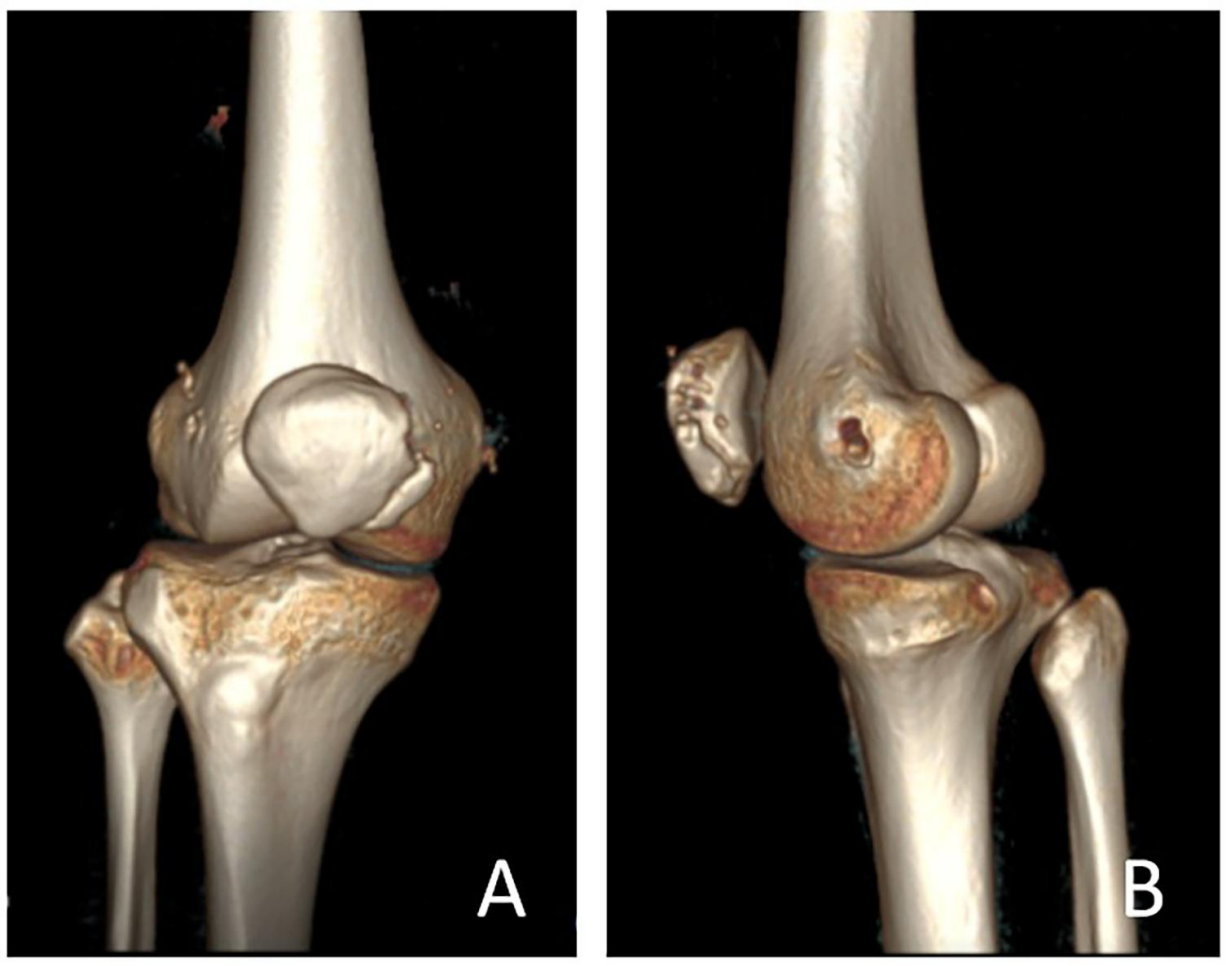
Six-month postoperative right knee CT scan with 3D reconstruction. Follow-up CT imaging and three-dimensional reconstruction at six months postoperatively demonstrate the right patella in normal anatomical position, indicating restoration of patellar alignment. **(A)** Frontal view. **(B)** Lateral view.

Radiographs of the right patella in anteroposterior and lateral axial views revealed a continuous cortical contour with a nodular high-density lesion adjacent to the patella, showing smooth margins consistent with BP (Figures 3A,B). The left patella appeared normal (Figure 3C). Computed tomography (CT) and three-dimensional reconstruction demonstrated lateral displacement of the right patella with a distinct high-density ossified nodule on the medial aspect of the joint (Figures 4, 5). Magnetic resonance imaging (MRI) further confirmed an elevated and slightly laterally displaced right patella, accompanied by a small nodular lesion showing short-T1 and short-T2 signals on the medial side (Figure 3D). No abnormal signal intensity was detected in the proximal tibia, fibula, or femur. The final diagnosis was BP with associated patellar elevation.

### 3.4 Complications

No severe complications were observed in the conservative treatment group. In the surgical treatment group, one patient developed a superficial wound infection that resolved after routine dressing changes and antibiotic therapy. Another patient experienced transient medial patellar hypoesthesia during the early postoperative period, which resolved spontaneously within three months. No major complications, including vascular or nerve injury, internal fixation failure, graft rupture, or epiphyseal damage, were observed.

## 4 Discussion

The patella, the largest sesamoid bone in the human body, is embedded within the quadriceps tendon and ossifies via secondary centers without primary ossification centers. In a small subset of children, two or more ossification centers may develop, potentially leading to the formation of a BP. Normally, these centers fuse into a single patella during puberty. Incomplete fusion, however, may result in one or two persistent subperiosteal bone fragments that remain throughout life, giving rise to bifid or trifid patellae. The incidence of BP is relatively low, ranging from 0.2% to 1.7%, with only approximately 2% of affected individuals exhibiting symptoms that require medical intervention (5, 6).

BP itself is not a disease entity but rather a developmental anomaly that alters the biomechanical properties of the patella. The fibrocartilaginous junction between the accessory ossification center and the main patella represents a mechanically vulnerable region (7). When patellar dislocation occurs, these two pathological conditions may mutually exacerbate each other. On one hand, during dislocation, the strong traction of the quadriceps and lateral impact forces are likely to act on this weak zone, resulting in stress-induced inflammation, edema, or even separation at the junction. Such pathological changes manifest clinically as pain and a locking sensation, marking the transition from an “asymptomatic” to a “symptomatic” stage [4]. On the other hand, the presence of BP—particularly Type III lesions located at the lateral epicondyle—may inherently compromise the integrity of medial stabilizing structures and alter the patellar tracking within the trochlear groove, thus serving as a potential trigger or aggravating factor for patellar instability (8, 9).

From a diagnostic perspective, a simple BP is frequently misinterpreted as a patellar fracture on plain radiographs. Key distinguishing features include smooth cortical margins with a sclerotic border, bilateral symmetry, and absence of traumatic history in BP. In contrast, patellar fractures typically present with sharp, irregular fracture lines, lack marginal sclerosis, are unilateral, and are associated with acute trauma. Cross-sectional imaging plays a crucial role in differential diagnosis. CT provides precise delineation of the bone structure, whereas MRI offers superior soft tissue characterization. MRI can clearly demonstrate the fibrocartilaginous connection between the accessory fragment and the main patella (appearing as high signal intensity on T2-weighted images, indicative of edema or inflammation) and simultaneously evaluate the morphology of the MPFL, articular cartilage, and meniscus. This multimodal assessment provides a comprehensive basis for individualized treatment planning (10). All pediatric patients in the present study underwent MRI evaluation to confirm the diagnosis and assess concomitant soft-tissue injury.

To date, two primary classification systems have been proposed for BP. The most widely used clinically is the Saupe classification (11), which categorizes BP based on the anatomical location of the accessory bone fragment: Type I (Lower Pole Type, ∼75%), with fragments at the inferior pole of the patella; Type II (Lateral Type, ∼20%), with fragments along the lateral border; and Type III (Upper Pole Type, ∼5%), with fragments at the superior pole. Although this classification remains commonly adopted, it has limitations in addressing medial BP or trifid patellae, as exemplified in some patient cases. To overcome these shortcomings, Oohashi et al. (5) proposed an updated classification that incorporates both the anatomical location and the number of accessory bone fragments, rendering it more comprehensive and applicable to all forms of BP, including bifid and tridentate variants.

The therapeutic approach was determined through a comprehensive evaluation of each patient's clinical presentation, imaging findings, and functional status. The central principle was a “dual-target strategy”, emphasizing the simultaneous management of patellar instability and symptomatic accessory ossification centers (12, 13). This individualized approach ensures both mechanical stability and resolution of pain associated with the BP, thereby optimizing long-term knee joint function and reducing recurrence risk.

In this study, the conservative treatment group primarily consisted of children presenting with initial dislocations and relatively stable patellar anatomy. The therapeutic goal was to facilitate the healing of acute soft-tissue injuries—including the medial patellofemoral ligament (MPFL) and the fibrocartilaginous interface between the bipartite fragments—through immobilization, while enhancing dynamic stability via strengthening of the vastus medialis oblique (VMO) muscle. However, our findings demonstrated that functional improvement in the conservative group was significantly inferior to that observed in the surgical cohort, with a recurrence rate of 27.8%. These results are consistent with previous reports indicating recurrence rates ranging from 15% to 50% following conservative management of patellar dislocation in adolescents (14, 15). This evidence suggests that in children with pre-existing BP—a condition characterized by inherent anatomical and biomechanical instability—conservative therapy alone is often insufficient to restore patellofemoral congruence or to prevent recurrent dislocation (11, 16).

The surgical group demonstrated significantly superior outcomes compared to the conservative treatment group, confirming the necessity of operative intervention addressing the underlying pathology (4). Our surgical strategy comprised three core components: (1) Collateral band release: This foundational procedure corrects lateral patellar tilt and alleviates excessive tension in the lateral soft tissues. (2) MPFL (17, 18) reconstruction: Considered the “gold standard” for restoring medial patellar stability, the MPFL serves as the primary static restraint against lateral patellar displacement and is frequently torn during dislocation events. Reconstruction of the MPFL effectively guides the patella back to its physiological trajectory. In pediatric patients, meticulous attention is given to tunnel placement to avoid damage to the growth plates (19–21). (3) Internal fixation of accessory bone fragments: For larger accessory fragments involving the articular surface, excision may compromise patellar biomechanics and joint function; in such cases, internal fixation is preferable. Screw compression fixation promotes bony union, effectively “reconstructing” the BP into a structurally normal patella and addressing the root cause of instability. In this study, all eight pediatric patients undergoing this procedure achieved complete bony union.

The statistical analysis conducted in this study provides strong support for our clinical observations. Among baseline characteristics, only the variable “dislocation type” differed between groups, reflecting the appropriateness of our grouping strategy based on disease severity rather than selective bias. Moreover, the significant differences in functional scores at final follow-up (*P* < 0.01) indicate that the improvements in knee function and pain relief observed in the surgical cohort were unlikely to be due to chance, and were markedly superior to those achieved with conservative treatment. These findings provide robust evidence to guide clinicians in formulating evidence-based treatment recommendations for families of pediatric patients with BP and patellar instability.

During MPFL reconstruction in cases of medial BP, surgeons should avoid selecting insertion sites on accessory bone fragments or spanning both the primary and accessory fragments. Except in exceptional circumstances, such as oversized accessory fragments, priority should be given to fixation on the primary bone fragment. This strategy not only minimizes physical obstruction but also ensures mechanical stability and surgical efficacy, making it a critical consideration in clinical practice. Future studies should aim to refine treatment protocols further. A thorough understanding of the clinical features, imaging characteristics, therapeutic strategies, and prognoses of medial BP associated with patellar subluxation will enhance diagnostic accuracy and inform optimal management of this condition.

Limitations: First, this study is a retrospective non-randomized controlled trial. Although we made every effort to ensure comparable baseline data between groups, selection bias may still exist. Second, the relatively small sample size (*n* = 42) is not insignificant in similar studies, but it lacks sufficient power for more detailed subgroup analyses (e.g., comparisons between different Saupe classifications and fixation methods). The imbalance in dislocation type between groups reflects real-world treatment selection but may have introduced selection bias. Finally, while the average 19.5-month follow-up duration can reflect short-to-medium term efficacy, the long-term impact on patellofemoral joint development in underage patients still requires extended observation periods.

## 5 Conclusion

Congenital BP combined with patellar dislocation represents a distinct and challenging condition in the pediatric population, requiring careful evaluation of both patellofemoral stability and the status of the accessory fragment. In this retrospective single-center study, both conservative and surgical management resulted in functional improvement; however, surgical treatment was associated with better functional scores, lower pain levels, and a reduced recurrence rate at short- to mid-term follow-up.

Importantly, the benefits of surgical intervention were most evident in selected pediatric patients presenting with recurrent dislocation, persistent symptoms, or clear anatomical abnormalities, particularly those related to unstable or symptomatic accessory bone fragments. Rather than introducing a novel concept of MPFL reconstruction in children, the present study emphasizes the importance of addressing symptomatic and anatomically unstable accessory fragments in conjunction with established patellofemoral stabilization procedures.

Given the retrospective design, limited sample size, and single-center nature of this study, these findings should be interpreted with caution. Further prospective studies with larger cohorts and longer follow-up are warranted to validate optimal treatment strategies for this uncommon condition.

## Data Availability

The original contributions presented in the study are included in the article/Supplementary Material, further inquiries can be directed to the corresponding author.
